# When to suspect a genetic syndrome

**DOI:** 10.1186/1824-7288-41-S2-A50

**Published:** 2015-09-30

**Authors:** Valentina Decimi, Luigi Memo

**Affiliations:** 1 Scuola di Specializzazione in Pediatria, Università di Milano Bicocca, Italy; 2 Unità Operativa Complessa di Pediatria e Patologia Neonatale, Ospedale San Martino, Belluno, Italy

## 

A genetic syndrome is a clinical situation that combines major and minor malformations, anomalies of growth and of psychomotor development .

Prompt recognition of features suggestive of genetic conditions can improve the selection of appropriate, cost-effective diagnostic tests, the performance of well-informed genetic counseling related to issues such as prognosis and future family planning and timely referral to subspecialists.

General themes that can alert pediatricians to the presence of genetic syndromes include dysmorphic features that are evident on physical examination; multiple anomalies in one patient; unexplained neurocognitive impairment; and a family history that is suggestive of a hereditary disease.

Taking an accurate three-generation family history is important when a genetic syndrome is suspected. Important elements include the age and sex of family members; when family members were affected by disease or when they died; the ethnic background; and if there is consanguinity.

Every effort should be made to obtain complete information about the gestation leading to the birth of the affected child. Events surrounding the birth of the child may be critical: antepartum status, length of labor, mode of delivery, baby's condition at birth, measurements.

Finally, information is obtained about the child's course since infancy along the lines of a standard pediatric history, but with a few special emphases: general health, growth, developmental progress, behavior, special testing or therapy.

The physical examination is of enormous importance: the basic techniques of physical diagnosis are used, but the examination is fine tuned to promote detection of many subtle physical clues that might otherwise be overlooked. Selected measurements of physical features can be extremely useful in confirming a clinical impression of abnormality.

The diagnosis of a particular disorder can be based on clinical features, laboratory data, or a combination of both.

The provision of an accurate diagnosis is only the first step in the long process of care for a child with genetic syndrome and the pediatrician may have a continuing role in working with the patient and the family.

